# Metabolic alkalosis in cystic fibrosis: from vascular volume depletion to impaired bicarbonate excretion

**DOI:** 10.3389/fendo.2024.1411317

**Published:** 2024-08-07

**Authors:** Manoocher Soleimani

**Affiliations:** ^1^ Department of Medicine, University of New Mexico, Albuquerque, NM, United States; ^2^ Research Services, New Mexico Veteran's Healthcare System, Albuquerque, NM, United States

**Keywords:** hypokalemia, Barter-like syndrome, diabetes mellitus, nephrotoxicity, acute kidney injury, kidney tubules

## Abstract

Cystic fibrosis (CF) is the most common life-threatening genetic disease in the United States and among people of European descent. Despite the widespread distribution of the cystic fibrosis transmembrane conductance regulator (CFTR) along kidney tubules, specific renal phenotypes attributable to CF have not been well documented. Recent studies have demonstrated the downregulation of the apical Cl^-^/HCO_3_^-^ exchanger pendrin (Slc26a4) in kidney B-intercalated cells of CF mouse models. These studies have shown that kidneys of both mice and humans with CF have an impaired ability to excrete excess HCO_3_^-^, thus developing metabolic alkalosis when subjected to excess HCO_3_^-^ intake. The purpose of this minireview is to discuss the latest advances on the role of pendrin as a molecule with dual critical roles in acid base regulation and systemic vascular volume homeostasis, specifically in CF. Given the immense prevalence of vascular volume depletion, which is primarily precipitated via enhanced chloride loss through perspiration, we suggest that the dominant presentation of metabolic alkalosis in CF is due to the impaired function of pendrin, which plays a critical role in systemic vascular volume and acid base homeostasis.

## Introduction

Cystic fibrosis (CF) is an autosomal recessive disorder caused by mutations in the cystic fibrosis transmembrane conductance regulator (CFTR), a cAMP/PKA-activated and ATP-regulated apical chloride channel that is widely expressed in epithelial tissues (1–3). Patients with CF are very prone to developing lung injury/infection, pancreatitis, and intestinal obstruction (1–3). While CFTR expression is detected in multiple nephron segments ranging from the proximal tubule to the thick ascending limb of Henle to the collecting duct, there is no discernible kidney phenotype attributed to CF. An original study in 2018 demonstrated that the HCO_3_^-^ secreting exchanger, pendrin, is downregulated in B-intercalated cells in CF mouse kidneys, making the animals prone to develop metabolic alkalosis in response to oral HCO_3_^-^ loading (4). More recent studies have confirmed and expanded on those findings by demonstrating the dysregulation of the kidney secretin receptor as a link between the oral bicarbonate load and the impaired renal HCO_3_^-^ excretion, thus promoting the development of metabolic alkalosis (5, 6). The lack of HCO_3_^-^ response to secretin (5, 6) is in complete agreement with the original studies in children with cystic fibrosis (7).

This mini review explores the pathogenesis of metabolic alkalosis in CF, specifically during vascular volume depletion, a phenomenon that is observed frequently in patients with CF. This can occur independent of HCO_3_^-^ loading and is primarily dependent on the severe loss of chloride ions through perspiration.

## Case presentation

A 24-year-old woman with CF was experiencing nausea, weakness, and general malaise, along with a decreased appetite for one week. She was admitted to the hospital following an abnormal lab test at an outpatient facility.

The patient had a BP of 100/60; PR of 82/min; BUN of 22 and serum creatinine of 1.2 mg/dl. The blood chemistry panel showed the following: Na^+^138 (reference 135-144), K^+^ 3.4 (reference 3.5 to 5.4), HCO_3_^-^ 33 (reference 22-29), and Cl^-^ 93 (reference 96-106) mEq/l.

A venous blood gas analysis showed: pH of 7.45 (reference for venous pH 7.32-7.42) and pCO_2_ of 45 mm Hg. The urine electrolytes were: Na^+^ 44, Cl^-^ 36, and K^+^ 35 meq/l. The above presentation is consistent with metabolic alkalosis (elevated serum HCO_3_^-^ concentration and blood pH values) along with respiratory compensation.

## Discussion

### Acid base abnormalities in CF

Although the primary acid base disorder in patients with CF is caused by impaired ventilatory gas exchanges leading to CO_2_ retention and chronic respiratory acidosis, the presence of metabolic alkalosis has been well documented in patients with CF for the last 5 decades (8–10). The preponderance of data has pointed to vascular volume depletion as the driving force in the generation of metabolic alkalosis in CF (8–10).

Recent studies have examined the pathogenesis of metabolic alkalosis in animal models and individuals with CF. These studies were mainly focused on acid base homeostasis in response to HCO_3_^-^ loading. The first study in 2018 demonstrated that kidneys of CF mice had an impaired ability to excrete excess HCO_3_^-^ during bicarbonate loading (4). In addition, there was a reduced expression of the HCO_3_^-^ secreting transporter, pendrin (Slc26a4), in the kidneys of CF mice (4). Separate experiments in CF mice showed impaired HCO_3_^-^ secretion in microperfused kidney collecting duct tubules (5). Follow up studies revealed that the kidney secretin receptor (Scrt) in the collecting duct is dysregulated in CF mice (5, 6), thus impairing the signal between the secretin secretion from the small intestine to a change in function of the kidney collecting ducts; and therefore, hampering the ability of the kidney to eliminate excess HCO_3_^-^ (5–7). Whether the expression levels of secretin receptor are decreased in kidneys of either CF mice or patients with CF remain unknown.

There are no studies that have comprehensively looked at those CF individuals who have not been subjected to an oral HCO_3_^-^ load, and yet present with metabolic alkalosis with increased frequency.

Published studies indicate that the two most common acid base disorders in cystic fibrosis are:

1. Chronic respiratory acidosis. This is presumed to be the most frequent acid base disorder owing to the impaired gas exchange at the alveolar level due to the damage caused by CF (8).
2. Metabolic alkalosis. Metabolic alkalosis is a frequent occurrence in adults with CF (8–13). Indeed, metabolic alkalosis could be the first manifestation of CF in toddlers and adolescents (9, 13).

The authors of the above study (8) concluded that metabolic alkalosis contributes to acute hypercapnic respiratory failure in adult CF individuals.

In a study examining acid base disorders in patients with chronic pulmonary dysfunction, the authors investigated acid base homeostasis in individuals with CF vs. chronic obstructive pulmonary disease (COPD) (12). The authors reported that in patients with comparable forced expiratory volume in 1 second (FEV1), majority of patients with CF, but not those with COPD, had metabolic alkalosis (12).

The analysis of acid base parameters in individuals with CF (12) revealed the presence of a primary metabolic alkalosis in 57% (8/14) of subjects, and a mixed metabolic alkalosis along with respiratory acidosis in 28% (4/14) of participants. The authors concluded that stable patients with CF lung are more prone to display metabolic alkalosis than in COPD patients (12).

In an original study, children in the Tucson area that had been diagnosed as having CF before the age of 12 months were examined to ascertain the prevalence of metabolic alkalosis as a major presenting manifestation of the disease (13). Five of eleven infants (46%) in whom CF had been diagnosed between 1 and 12 months of age were initially seen with metabolic alkalosis, hypokalemia, and hypochloremia, unassociated with major pulmonary and/or gastrointestinal symptoms. Two infants had repeated episodes of metabolic alkalosis; for one of these infants, both episodes of metabolic alkalosis occurred before the diagnosis of CF (13). Ground breaking studies had convincingly demonstrated that individuals with cystic fibrosis could lose vast amounts of salt (Cl^-^ and Na^+^) in sweat, specifically in hot weather (14–16), which would precipitate vascular volume depletion. The enhanced loss of salt through sweat in individuals with CF was shown to be primarily due to a defective route of transcellular Cl^-^ uptake in sweat duct epithelium (17). It was postulated that chronic loss of sweat electrolytes together with mild gastrointestinal or respiratory illness may predispose young individuals with CF to develop a severe electrolyte and acid-base disturbance (13). Taken together the above studies indicate that metabolic alkalosis is a frequent occurrence in cystic fibrosis and may be independent of the presence of lung damage or infection.

### Cystic fibrosis and Pseudo Barter (Bartter-Like) syndrome

The excess loss of chloride (salt) through sweat (CF), gastric content (vomiting), or kidney (in individuals with Bartter Syndrome or treated with loop diuretics) is a well-known factor that results in vascular volume depletion and precipitates metabolic alkalosis (18–20). Unlike patients who suffer from gastric acid loss (vomiting) and present with volume depletion and a very low urine chloride, patients with Bartter Syndrome present with volume depletion, metabolic alkalosis, and increased urine chloride (18, 21). Patients with CF can exhibit volume depletion and increased (but not decreased) urine chloride, a phenomenon which has been referred to as Pseudo Bartter Syndrome (22). It should be noted that in severe instances of volume depletion, patients with CF could present with a low urine chloride consistent with enhanced absorption of chloride in more proximal nephron segments (11).

### CFTR and kidney collecting duct

The expression of CFTR in collecting duct cells varies according to cell type. It is most abundantly expressed in HCO_3_^-^ secreting B-intercalated cells, followed by principal cells, and with the lowest levels found in acid-secreting A-intercalated cells (23). In an original study in 2018, the expression of the apical Cl^-^/HCO_3_^-^ exchanger, pendrin, was found to be significantly reduced, which resulted in impaired HCO_3_^-^ excretion in response to an oral HCO_3_^-^ load (4). These studies were confirmed by other investigators (5, 6). It was concluded that patients with CF are prone to the development of metabolic alkalosis when subjected to oral HCO_3_^-^ loading secondary to the inactivation of the bicarbonate secreting transporter, pendrin in their kidneys (4–6). Additional investigations have shed more light on the link between oral HCO_3_^-^ loading and the impaired ability of the CF kidney to eliminate excess HCO_3_^-^ (5, 6). These experiments conducted in CF mice demonstrated dysregulation of Scrt (secretin receptor) in kidney B-intercalated cells (6). Taken together, these studies indicated that SCTR plays an important role in linking the circulating secretin with pendrin activation in kidney B-intercalated cells (5, 6).

### Cystic fibrosis, vascular volume depletion, and pendrin

Pendrin plays a dual role in acid-base and vascular volume regulation by secreting HCO_3_^-^ into the lumen of the collecting duct in exchange for absorbing chloride (24–32). As such, pendrin is critical to both vascular volume regulation and acid base homeostasis (28–32). Increased circulating aldosterone, either as a primary event or consequent to vascular volume depletion, increases pendrin function in part through its translocation to the apical membrane from the subapical region (28, 30–32). In pendrin-deficient mice, imposing vascular volume depletion was associated with the inability to absorb chloride and secret HCO_3_^-^, thus resulting in the generation of metabolic alkalosis (28, 30–33). In a remarkably similar manner, CF mice developed a significantly higher serum HCO_3_^-^ concentration vs. wildtype (WT) animals when placed on a salt deficient diet (with the serum HCO_3_^-^ concentration increasing to 29.2 mEq/L in CF mice vs. 26.7 in WT; p<0.03) further supporting the critical role that pendrin plays in both volume regulation and acid base balance (4).

**Figure 1:**
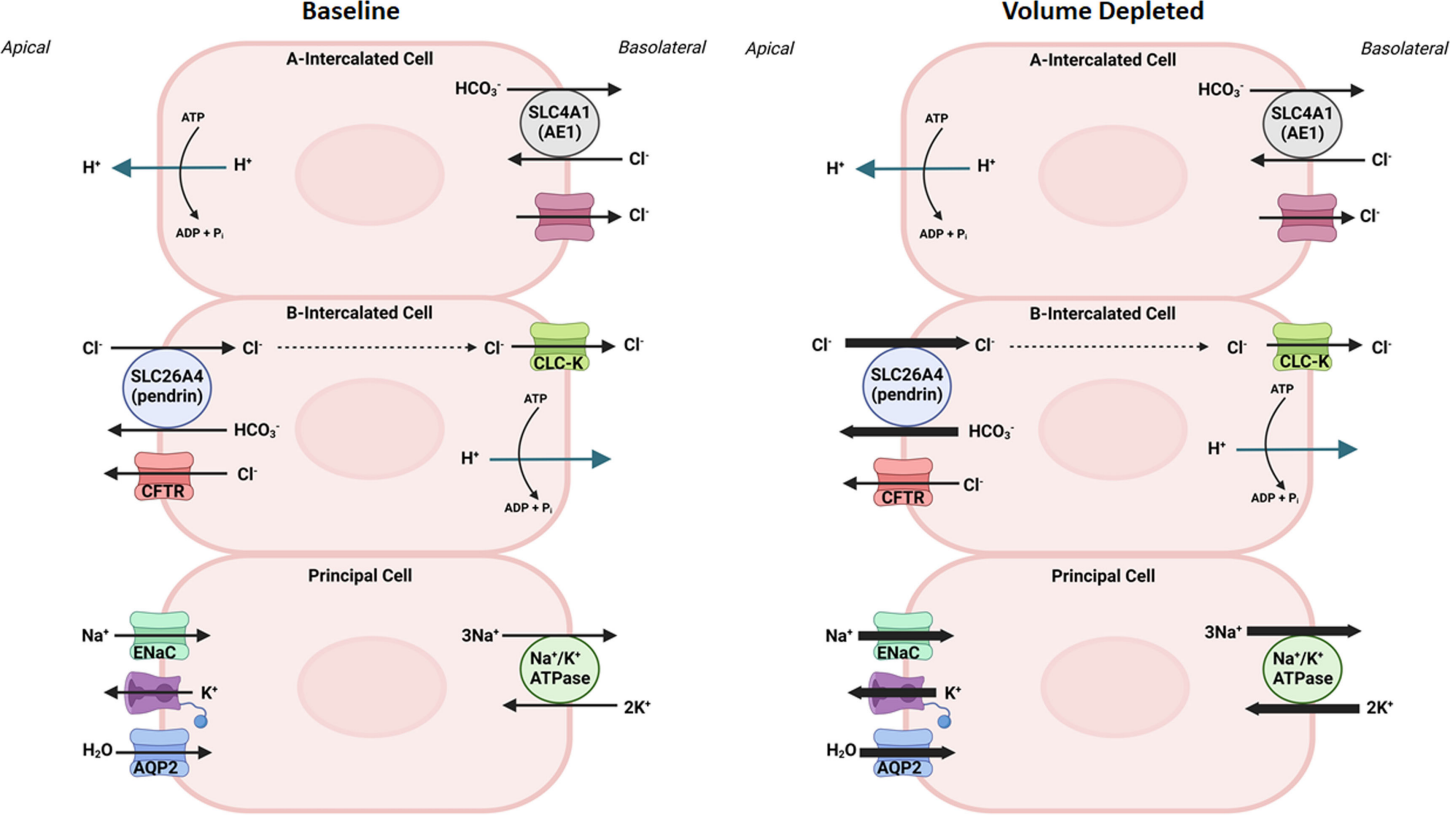
The role of collecting duct cells in acid base and vascular volume homeostasis. Left panel, is a schematic diagram indicating acid (H^+^) and base (HCO_3_^-^) secretion in A- and B- intercalated cells, and electrolyte and water transport in principal cells in kidney collecting duct cells at baseline state. Right panel is a schematic diagram depicting the activation of pendrin in chloride absorption and HCO_3_^-^ secretion in the kidney B-intercalated cells, and ENaC in sodium absorption in principal cells in volume depleted states.

**Figure 1**, left panel, is a schematic diagram depicting acid (H^+^) and base (HCO_3_^-^) secretion in A and B intercalated cells, respectievely, and electrolyte and water transport in principal cells in kidney collecting duct cells. As indicated in **Figure 1**, right panel, pendrin mediates chloride absorption and HCO_3_- secretion in B intercalated cells and shows overactivation in volume depleted state. As such, the impaired ability of pendrin to absorb luminal chloride and secret HCO_3_^-^ results in an excessive loss of chloride into urine in the setting of vascular volume depletion, thus worsening the magnitude of alkalosis (Pseudo Bartter Syndrome).

### The role of CFTR in epithelial physiology

Cystic fibrosis affects the integrity and functional properties of epithelial cells of several organs, including the respiratory tract, exocrine pancreas, intestine, vas deferens, hepatobiliary system, and exocrine sweat glands.

Published studies indicate that:

1. CFTR plays a critical role in regulating luminal pH and maintaining epithelial surface hydration (34, 35).
2. The activation of CFTR enhances HCO_3_^-^ secretion across the luminal membrane in airway epithelia, pancreatic ducts, intestines, and several other epithelial tissues (1–3, 34–41). It was suggested that bicarbonate transport mediated via an anion exchange mechanism may be defective in tissues from individuals with cystic fibrosis (42). A critical discovery revealed that in addition to a defective Cl^-^ transport, CFTR mutations disrupt the transport of HCO_3_^-^ in various tissues in cystic fibrosis (43), highlighting the pathophysiological role of impaired HCO_3_^-^ secretion in cystic fibrosis (44).
3. Luminal pH plays an important role in epithelial barrier function and innate defense, particularly in the airways and GI tract (34, 35, 40, 41, 45).

### The role of SLC26 family of Cl^-^/HCO_3_^-^ exchangers in CFTR-activated HCO_3_^-^ secretion in epithelial tissues

**Figure 2:**
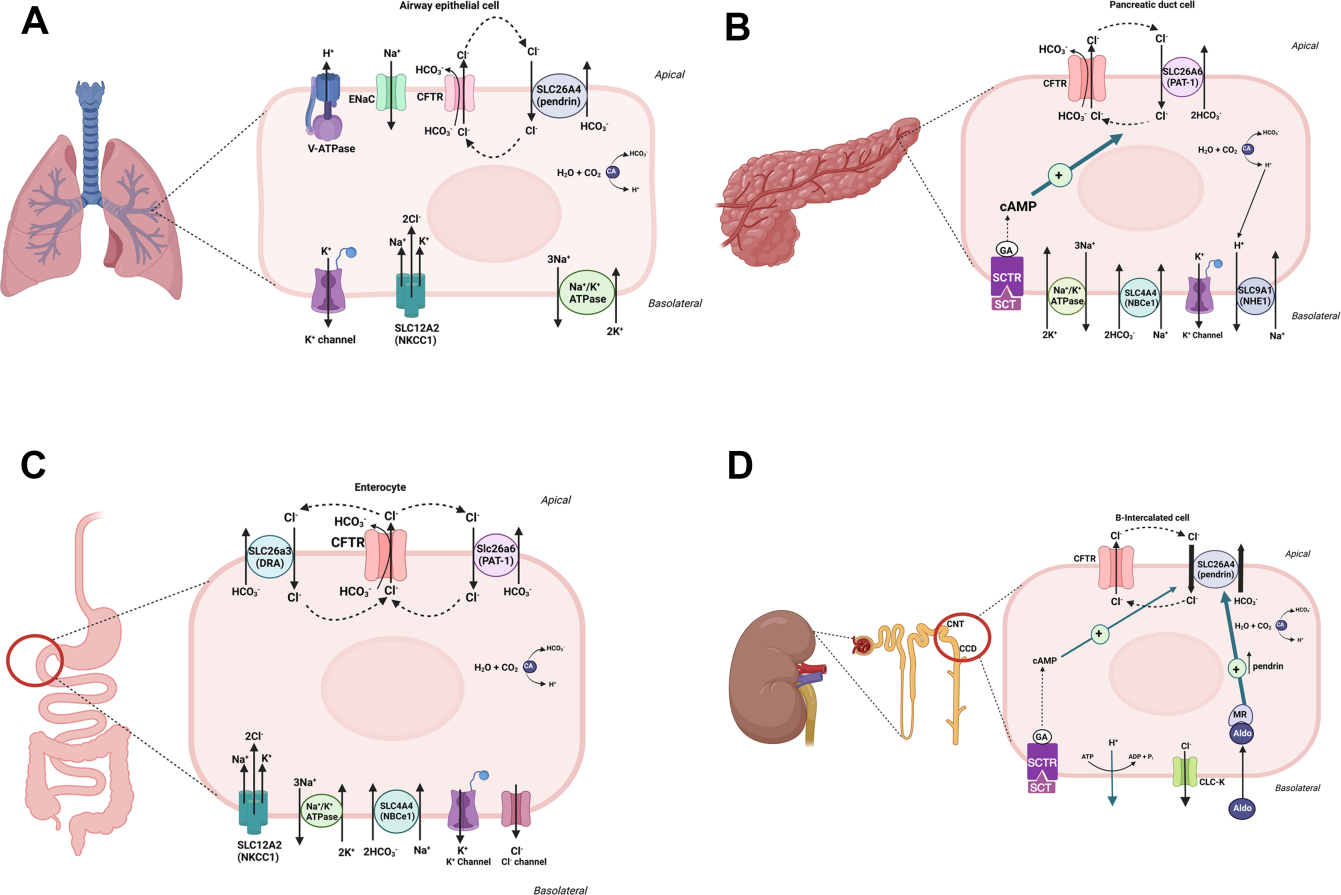
A schematic diagram depicting CFTR-dependent HCO_3_^-^ secretion in alveolar cells **(A)**, pancreatic duct **(B)**, small intestine **(C)** and kidney B-intercalated cells **(D)**. As noted, CFTR, when activated, may become permeant to both Cl^-^ and HCO_3_^-^ ions in alveolar, pancreatic duct and enterocytes. In kidney B intercalated cells, the role of pendrin in absorbing chloride and secreting HCO_3_^-^ is paramount. The roles of aldosterone, which is critical in pendrin translocation and activation in volume depleted states, and the SCTR, which links secretin to pendrin-mediated HCO_3_^-^ secretion are highlighted in kidney B-intercalated cells **(D)**.

The activation of CFTR in pancreatic duct cells, airway epithelia, and the intestine enhances HCO_3_^-^ secretion. Intense research on the role of CFTR activation in HCO_3_^-^ secretion has identified several apical Cl^-^/HCO_3_^-^ exchangers from the anion transporting SLC26 family that physically interact and function in tandem with CFTR, thus contributing to enhanced HCO_3_^-^ transport into the lumen of epithelial cells (46, 47). These apical Cl^-^/HCO_3_^-^ exchangers include SLC26A3 (DRA), SLC26A4 (pendrin) and SLC26A6 (PAT1) (48–50) and interact with the CFTR molecule through their STAS domain (46–49). The schematic diagram in **Figure 2** depicts a remarkable similarity between the molecular machineries that facilitate HCO_3_^-^ and Cl^-^ secretion in response to CFTR activation in airway epithelia (A), pancreatic ducts (B), and enterocytes of small intestine (C). It is plausible that the kidney B-intercalated cells may exhibit a similar activating interaction between CFTR and the Cl^-^/HCO_3_^-^ exchanger, pendrin (**Figure 2D**). As noted, CFTR mutations result in a widespread HCO_3_^-^ secretion defect in the epithelial cells of the airways, pancreatic ducts, intestines, and the kidney collecting duct (4–6, 34–45). In addition to interacting with and stimulating the SLC26 Cl^-^/HCO_3_^-^ exchangers, a large number of studies support the conclusion that CFTR, when activated, becomes permeant to both Cl^-^ and HCO_3_^-^ (reviewed in 51). Recent publications indicate that permeability of CFTR to various anions can be modulated by the WNK-SPAK pathway (51). The schematic diagrams in **Figures 2A–C** depicts the dual functional roles of CFTR in transporting Cl^-^ and HCO_3_^-^ ions in an activated state in the pancreatic duct, enterocytes, and alveolar cells.

### Cystic fibrosis, pendrin and oral HCO_3_^-^ load

Excess HCO_3_^-^ intake in WT mice results in an enhanced expression of pendrin along with increased HCO_3_^-^ excretion (26, 27). A similar maneuver in CF mice, which display significant downregulation of pendrin, resulted in impaired HCO_3_^-^ excretion along with the development of metabolic alkalosis (4–6, 36). These results indicate that a functional pendrin plays a sizeable role in preventing the generation of metabolic alkalosis in response to oral HCO_3_^-^ loading. Based on the critical role of SCTR in activating pendrin-mediated HCO_3_^-^ secretion in kidney B-intercalated (B-IC) cells in response to oral base (e.g., HCO_3_^-^) loading, recent studies suggest that loss of SCTR in CF impairs the appropriate increase of renal base excretion during acute base loading and that SCTR is necessary for rapid correction of metabolic alkalosis (5, 36). Extrapolated from these studies (5–7), it was proposed that the prevalent metabolic alkalosis in patients with CF could be explained by the absence of secretin-induced urinary HCO_3_^-^ excretion (36). It was further suggested that patients with CF could be given a urine pH test after oral HCO_3_^-^ loading to validate the efficacy of CFTR modulators in treating cystic fibrosis. Recent studies point to the impaired ability of CF patients to increase their urine HCO_3_^-^ excretion during the presence of metabolic alkalosis (52).

It should be noted that while CF mice do not recapitulate several phenotypic presentations of individuals with CF (such as airway defect or male infertility), the GI and kidney manifestations in CF mice mirror those defects in human CF. In a search for non-primate models of CF, studies show that the porcine CF model exhibits a strong phenotypic resemblance to humans with CF in that the porcine model shows bacterial colonization, as well as airway anomalies at birth and the impaired ability of CF piglets to clear bacteria (53, 54). It would be enlightening to examine the role of pendrin and HCO_3_^-^ secretion pathways in kidneys of porcine CF models. This issue becomes critical because it can shed light on whether lung infection/injury plays any role in the generation of metabolic alkalosis in cystic fibrosis.

As discussed in this minireview, the major determinant of metabolic alkalosis generation in CF is the development of vascular volume depletion, a process that is unencumbered by and independent of oral HCO_3_^-^ load (8–13). It should further be noted that due to multiple medical ailments, patients with CF may exhibit a false positive response (impaired excretion of HCO_3_^-^) to the oral base loading test due to CF-independent tubular abnormalities. Amongst these medical maladies are:

1. Frequent lung infections that require multiple and aggressive antimicrobial therapy, which can impact kidney tubular function and the glomerular filtration rate (GFR).
2. Development of pancreatic insufficiency and diabetes mellitus, which can lead to malabsorption in patients with CF.
3. Moreover, patients with CF are at an increased risk for acute kidney injury due to the use of nephrotoxic agents, development of vascular volume depletion, or both, which can impair the physiological functions of kidney tubules and produce false positive (impaired HCO_3_^-^ excretion) results when testing for HCO_3_^-^ excretion.
4. Severe vascular volume depletion and the resulting decreased kidney perfusion can interefer with excess HCO_3_^-^ excretion in individuals subjected to enhanced base load, whether or not they have CF.
5. Furthermore, known electrolyte abnormalities such as hypokalemia consequent to vascular volume depletion or due to the development of hypomagnesemia –either secondary to malabsorption or aminoglycoside-induced pseudo–Bartter Syndrome (14, 55–57)- can decrease pendrin expression and impair HCO_3_^-^ excretion following base loading.
6. Lastly, pharmaceutical agents such as carbonic anhydrase inhibitors can downregulate kidney pendrin in a CF-independent manner (55).

Taken together, there are multiple medical maladies, as listed above, that are independent of CF that can interfere with enhanced excretion of oral HCO_3_^-^ load.

### Conclusion

In the kidney collecting duct, the Cl^-^/HCO_3_^-^ exchanger, pendrin (SLC26A4), significantly contributes to the maintenance of systemic vascular volume and blood pH during vascular volume depletion by absorbing luminal chloride in exchange for HCO_3_^-^ secretion. This important defensive mechanism is hampered in individuals with CF, thus making them prone to the development of metabolic alkalosis and worsening of vascular volume depletion by excessive loss of salt in their kidneys (Pseudo Bartter Syndrome). In addition, kidneys of patients with CF may be ill-equipped to unload excess blood HCO_3_^-^ concentration via pendrin due to the impaired cross talk between the circulating secretin and kidney B-intercalated cells. Please see the schematic diagram in kidney B-intercalated cells (**Figure 2D**). While the optimal treatment for the correction of metabolic alkalosis would be via the rectification of the underlying defect in the CFTR, the treatment of systemic volume depletion will go a long way to ameliorate existing metabolic alkalosis in patients with CF.

## Author contributions

MS: Conceptualization, Formal analysis, Funding acquisition, Writing – original draft, Writing – review & editing.
